# Short-Term Genome Stability of Serial *Clostridium difficile* Ribotype 027 Isolates in an Experimental Gut Model and Recurrent Human Disease

**DOI:** 10.1371/journal.pone.0063540

**Published:** 2013-05-15

**Authors:** David W. Eyre, A. Sarah Walker, Jane Freeman, Simon D. Baines, Warren N. Fawley, Caroline H. Chilton, David Griffiths, Alison Vaughan, Derrick W. Crook, Tim E. A. Peto, Mark H. Wilcox

**Affiliations:** 1 NIHR Oxford Biomedical Research Centre, John Radcliffe Hospital, Oxford, Oxfordshire, United Kingdom; 2 Leeds Institute of Molecular Medicine, University of Leeds, Leeds, West Yorkshire, United Kingdom; Robert Koch Institut, Germany

## Abstract

**Background:**

*Clostridium difficile* whole genome sequencing has the potential to identify related isolates, even among otherwise indistinguishable strains, but interpretation depends on understanding genomic variation within isolates and individuals.

**Methods:**

Serial isolates from two scenarios were whole genome sequenced. Firstly, 62 isolates from 29 timepoints from three *in vitro* gut models, inoculated with a NAP1/027 strain. Secondly, 122 isolates from 44 patients (2–8 samples/patient) with mostly recurrent/on-going symptomatic NAP-1/027 *C. difficile* infection. Reference-based mapping was used to identify single nucleotide variants (SNVs).

**Results:**

Across three gut model inductions, two with antibiotic treatment, total 137 days, only two new SNVs became established. Pre-existing minority SNVs became dominant in two models. Several SNVs were detected, only present in the minority of colonies at one/two timepoints. The median (inter-quartile range) [range] time between patients’ first and last samples was 60 (29.5–118.5) [0–561] days. Within-patient *C. difficile* evolution was 0.45 SNVs/called genome/year (95%CI 0.00–1.28) and within-host diversity was 0.28 SNVs/called genome (0.05–0.53). 26/28 gut model and patient SNVs were non-synonymous, affecting a range of gene targets.

**Conclusions:**

The consistency of whole genome sequencing data from gut model *C. difficile* isolates, and the high stability of genomic sequences in isolates from patients, supports the use of whole genome sequencing in detailed transmission investigations.

## Introduction

Multiple typing and fingerprinting schemes have been described for the common healthcare-associated pathogen *Clostridium difficile*. These have variable discriminatory power and practicality: particularly regarding utility by non-specialist laboratories, availability of reference databases and cost. [1] We have recently successfully applied sequence-based typing (multi-locus sequence typing, MLST)[2]–[4] to the investigation of *C. difficile* transmission. [5] This approach showed that the majority of 1282 *C. difficile* infection (CDI) cases over 2.5 years could not be explained by recent hospital ward contact with other symptomatic cases. However, the dominance of epidemic *C. difficile* types in many scenarios, for example ribotype 027 (NAP-1), [6], [7] emphasises the need for typing methods with high discriminatory power that can be applied in multiple settings.

The increasing availability and affordability of next-generation whole genome sequencing (WGS) technology offers much greater genetic resolution that has hitherto been available.[8]–[10] However, increased discriminatory power may be undermined by genetic instability. There is therefore a key need to understand the stability of the *C. difficile* genome over time (ie its microevolution) in order to maximise the potential value of WGS, since plausibility of transmission between closely related isolates can only be interpreted in the context of within-patient diversity at single points in time, and over time. Ideally, such studies should use data obtained from both controlled experimental and *in vivo* settings to test the reproducibility of observations. Crucially, such combined approaches offer the potential to examine the effects of interventions/exposures. We have therefore investigated *C. difficile* genome stability from successive samples recovered from a gut model that simulates CDI and its treatment, [11], [12] and from serial isolates recovered from patients with on-going or recurrent CDI.

## Materials and Methods

**Figure 1:**
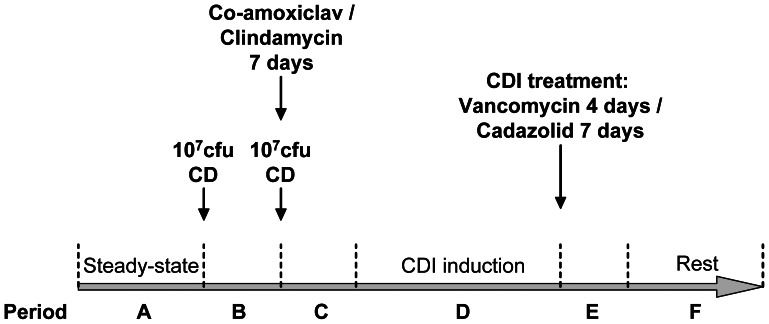
Gut model samples. A schematic diagram of the gut model experiments to induce and treat *Clostridium difficile* infection. CD, *Clostridium difficile*. CDI, *C. difficile infection*. Cfu, colony forming units.

**Figure 2:**
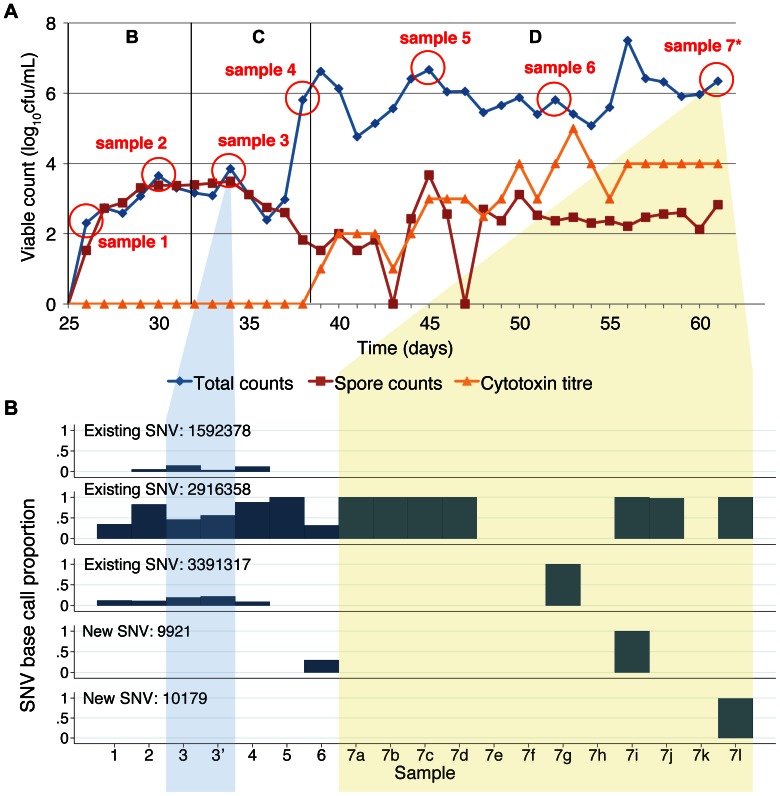
Co-amoxiclav induction gut model samples and variable site base counts. Panel A shows the total viable counts, spore counts and cytotoxin titres. Sampling points are shown as circles. Sampling points where 12 colonies were sub-cultured and sequenced separately are shown with an *. Panel B shows the base counts obtained at the variable sites identified between sequences. Each sample is represented in a different column. Samples taken at different timepoints are numbered sequentially. Where 12 colonies were sub-cultured at a given timepoint these are indicated by different letters following the sample number and yellow background shading. Replicate samples sequenced to assess the accuracy of the sequencing are shown with a‘, e.g. 3’ and blue background shading. The proportion of high quality (base quality PHRED scaled score ≥30, mapping quality ≥30) base counts supporting the SNV identified is shown for each variable site as a separate row. Median (range) calls per site 49 (23–99). No site with >2 different base calls.

**Figure 3:**
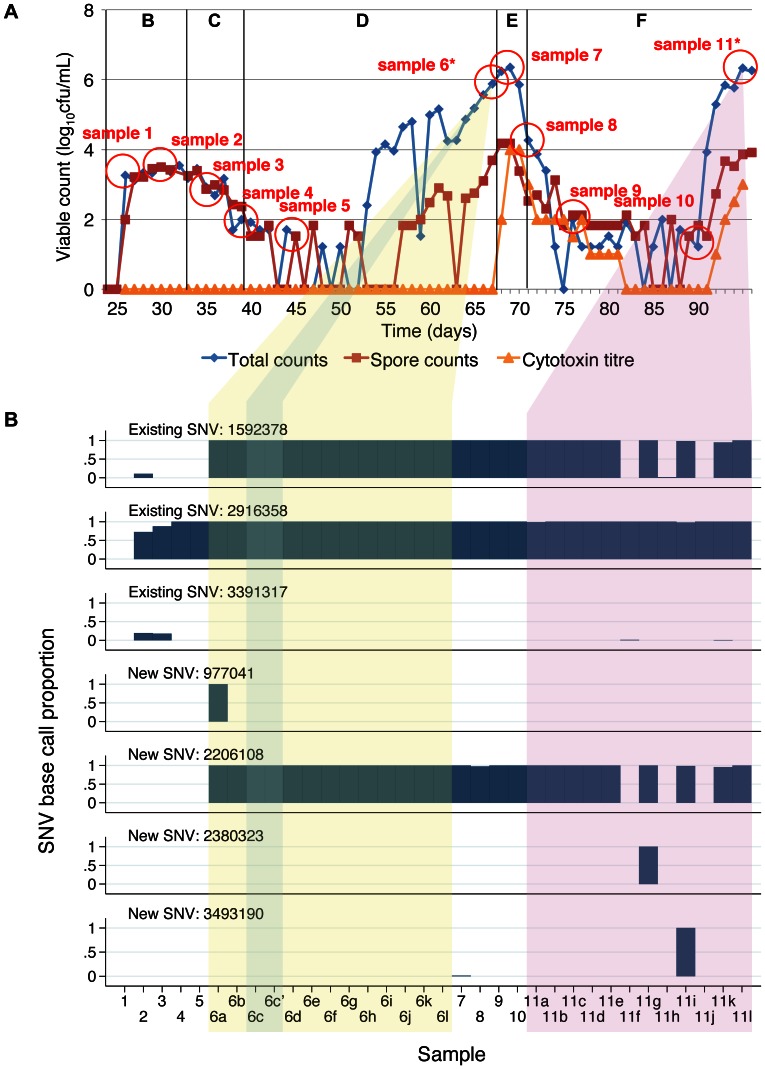
Clindamycin-induction, vancomycin-treatment gut model samples and variable site base counts. Panel A shows the total viable counts, spore counts and cytotoxin titres. Sampling points are shown as circles. Sampling points where 12 colonies were sub-cultured and sequenced separately are shown with an *. Panel B shows the base counts obtained at the variable sites identified between sequences. Each sample is represented in a different column. Samples taken at different timepoints are numbered sequentially. Where 12 colonies were sub-cultured at a given timepoint these are indicated by different letters following the sample number and yellow background shading (pre-treatment) and purple background shading (post-treatment). Replicate samples sequenced to assess the accuracy of the sequencing are shown with a‘, e.g. 6c’ and blue background shading. The proportion of high quality (base quality ≥30, mapping quality ≥30) base counts supporting the SNV identified is shown for each variable site as a separate row. Median (range) calls per site 71 (28–198). No site with >2 different base calls.

**Figure 4:**
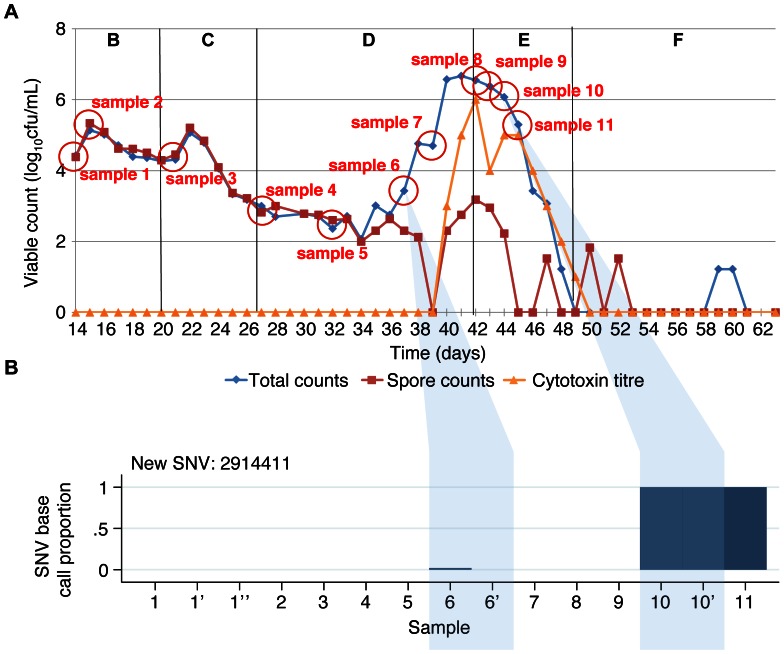
Clindamycin-induction, cadazolid-treatment gut model samples and variable site base counts. Panel A shows the total viable counts, spore counts and cytotoxin titres. Sampling points are shown as circles. Panel B shows the base counts obtained at the variable sites identified between sequences. Each sample is represented in a different column. Samples taken at different timepoints are numbered sequentially. Replicate samples sequenced to assess the accuracy of the sequencing are shown with a‘, e.g. 6’ and blue shading. The proportion of high quality (base quality ≥30, mapping quality ≥30) base counts supporting the SNV identified is shown for each variable site as a separate row. Median (range) calls per site 57 (28–89). No site with >2 different base calls.

We collected *C. difficile* isolates from each of the following two distinct sources. Firstly, serial samples were taken for whole genome sequencing from 3 human gut CDI model experiments conducted to investigate specific research questions.[12]–[14] One model simulated CDI resulting from co-amoxiclav exposure (without CDI treatment), and the other two, CDI following clindamycin exposure, then subsequently treated either with vancomycin for 4 days or cadazolid (a novel oxazolidinone/quinolone hybrid [15]) for 7 days. The gut model used is described in full elsewhere,[12]–[14] however, briefly, it consists of three vessels in a weir cascade system, held under anaerobic conditions, and designed to reflect the increasing alkalinity (pH 5.5–6.8) and reduced substrate availability of the proximal to distal colon. In each experiment the system was inoculated with a pooled faecal slurry of *C. difficile* culture-negative faeces that were obtained from five healthy (*C. difficile* negative) elderly (≥60y) volunteers. The model was top-fed with a growth medium at a controlled rate. The following time periods key time periods were observed (Figure 1): Period A, steady state, when the model was left to equilibrate for at least two weeks prior to interventions; Period B, *C. difficile* spore inoculation, when the model was instilled with ∼10^7^ cfu *C. difficile* ribotype 027 spores and monitored for seven days; Period C, *C. difficile* spore inoculation and CDI initiation, when the model was instilled with a further ∼10^7^cfu *C. difficile* ribotype 027 spores and CDI-provoking antibiotics (co-amoxiclav 8 mg/L, TID, 7d; or clindamycin, 33.9 mg/L, QID, 7d); Period D, CDI, i.e. *C. difficile* cytotoxin induction, following provocative antibiotic treatment; Period E, In two of the three gut models, CDI treatment (vancomycin 125 mg/L, QID 4d, or cadazolid 250 mg/L BID 7d [14], [15]) following high level cytotoxin production (≥4 relative units) for ≥2 days; and finally period F, a rest period when no further interventions took place. *C. difficile* isolates were obtained from the gut model at the timepoints indicated in Figures 2, 3, and 4, chosen to represent the different stages of *C. difficile* evolution within the gut and the variation in antibiotic selection pressure. Following culture on Columbia blood agar (ANO_2_, 24 h, 37°C). DNA was extracted from sub-culture of a representative sweep taken across the primary culture plate, except for 3 occasions where 12 individual colonies were sub-cultured and DNA extracted from the resulting growth to assess for within-‘host’ diversity (co-amoxiclav model, final timepoint and clindamycin/vancomycin model immediately prior to CDI treatment and at the final timepoint).

Secondly, we analysed whole genome sequences from *C. difficile* cultured from 44 patients sampled more than once during on-going or recurrent CDI due to *C. difficile* ribotype 027 (multi-locus sequence type 1, ST1). These CDI cases were identified from sequential unformed faecal samples that had been submitted to the Oxford University Hospitals NHS Trust Microbiology laboratory between September 2006 and March 2011 for routine investigation of microbial causes of diarrhoea. The cases build upon an existing collection of whole genome sequences from serially sampled patients which included 28 ribotype 027 infections which were previously analysed together with 63 other samples. [16] *C. difficile* toxin enzyme immunoassay (Meridian Biosciences, Cincinnati, OH) positive samples were cultured following an alcohol-shock on modified Brazier’s cycloserine-cefoxitin-egg yolk agar. [2] Culture positive isolates underwent MLST. [2] DNA was extracted from sub-culture of a single colony from each positive clinical sample.

DNA samples were prepared for sequencing using standard Illumina (San Diego, California, USA) and adapted protocols. Pools of 96 samples were sequenced at the Wellcome Trust Centre for Human Genetics, Oxford, UK, using sequencing-by-synthesis technology, [17] on the Illumina Genome Analyzer II (GAII), GAIIx, and HiSeq platforms, generating 50–108 base pair reads. Sequence reads were mapped using Stampy v1.0.11 (without Burrows-Wheeler Aligner pre-mapping, using an expected substitution rate of 0.01) [18] to a ribotype 027 *C. difficile* reference genome, CD196 (Genbank: FN538970) [19], to produce BAM files used in subsequent base-calling. Single nucleotide variants (SNVs) were identified across all mapped non-repetitive sites using SAMtools (version 0.1.12–10) [20] mpileup with the extended base-alignment quality flag, after parameter tuning based on bacterial sequences (options ‘-E -M0 -Q30 -q30 -m2 -D –S’). To identify repetitive regions, BLAST [21] searches of the CD196 reference genome were made using fragments of the same genome. As all strains analysed in this study were the same lineage as the reference strain (ribotype 027), we did not mask mobile elements. We only used SNVs that were supported by ≥5 reads, including one in each direction. A consensus of ≥75% was also required to support a SNV, and calls had to be homozygous under a diploid model.

As a quality control, five isolates had DNA extracted twice, and were sequenced twice, producing indistinguishable final sequences. The within-host diversity and rate of *C. difficile* evolution was estimated by maximum likelihood from first and last isolates from serially sampled patients under a coalescent model [22] assuming a Poisson distribution for the accumulation of mutations (see Text S1). Confidence intervals were obtained by parametric bootstrap. dN/dS ratios were calculated using the Nei and Gojobori method. [23] Genomes were sampled with replacement 1000 times to generate bootstrap confidence intervals.

Ethical approval for use of patient samples without individual consent was provided by the Oxford Research Ethics Committee (10/H0505/83) and the National Information Governance Board (8-05(e)/2010).

## Results

### Genomic Stability of C. difficile Gut Model Isolates

A total of 62 *C. difficile* isolates were obtained and successfully sequenced from 29 samples collected from 3 gut models: 18 isolates from the co-amoxiclav induction model (7 timepoints over 35 days, 12 isolates at last timepoint, figure 2), 33 isolates from the clindamycin induction, vancomycin treatment model (11 timepoints over 70 days, 12 isolates at two timepoints, just prior to, and following vancomycin treatment, figure 3), and 11 isolates from the clindamycin/cadazolid model (11 timepoints over 32 days, figure 4). A mean 90.3% of the CD196 reference genome was called, with a mean read depth of 62.3.

A total of 10 variable sites were identified across all 3 gut models (figures 2, 3, and 4). Both the co-amoxiclav and clindamycin-vancomycin models were inoculated with the same *C. difficile* ribotype 027 isolate. Sequences obtained from whole culture plate sweeps at the first 2 timepoints in these models show evidence of heterozygousity at 3 of the variant sites in both experiments (sites 1592378, 2916358, 3391317) suggesting that a mixture of *C. difficile* with and without these SNVs was present in the initial inoculum. In the co-amoxiclav model one of these SNVs (2916358) persisted throughout and was present in 7 of the 12 colonies sequenced at the final timepoint. In contrast the proportion of reads containing the other 2 SNVs diminished during the population expansion associated with CDI cytotoxin induction (phase D), and were present in 0/12 and 1/12 colonies at the end of the model. In the clindamycin/vancomycin model, 2 of the pre-existing SNVs became established (sites 1592378, 2916358), as well as a new SNV which arose during induction and persisted during treatment (2206108). In both models, a number of minority variants emerged, evidence of which could be seen in multiple picks taken following CDI cytotoxin induction (co-amoxiclav model, sites 9921 and 10179; clindamycin/vancomycin model, site 977041) and CDI treatment (clindamycin/vancomycin model, 2380323, 3493190) (figures 2b, 3b). In the final model, clindamycin/cadazolid, a single SNV was observed at the final 2 timepoints (2914411) following treatment.

Overall, over a total 137 days of follow up, including three CDI inductions and two CDI treatments, only two new SNVs emerged that became established in the majority of sequences. Pre-existing SNVs present in the minority of the within-model inoculum population became dominant twice in one model, and once in another. Five additional SNVs were detected that were only present in the minority of colonies at one or two timepoints.

### Genomic Stability of C. difficile Patient Isolates

**Figure 5:**
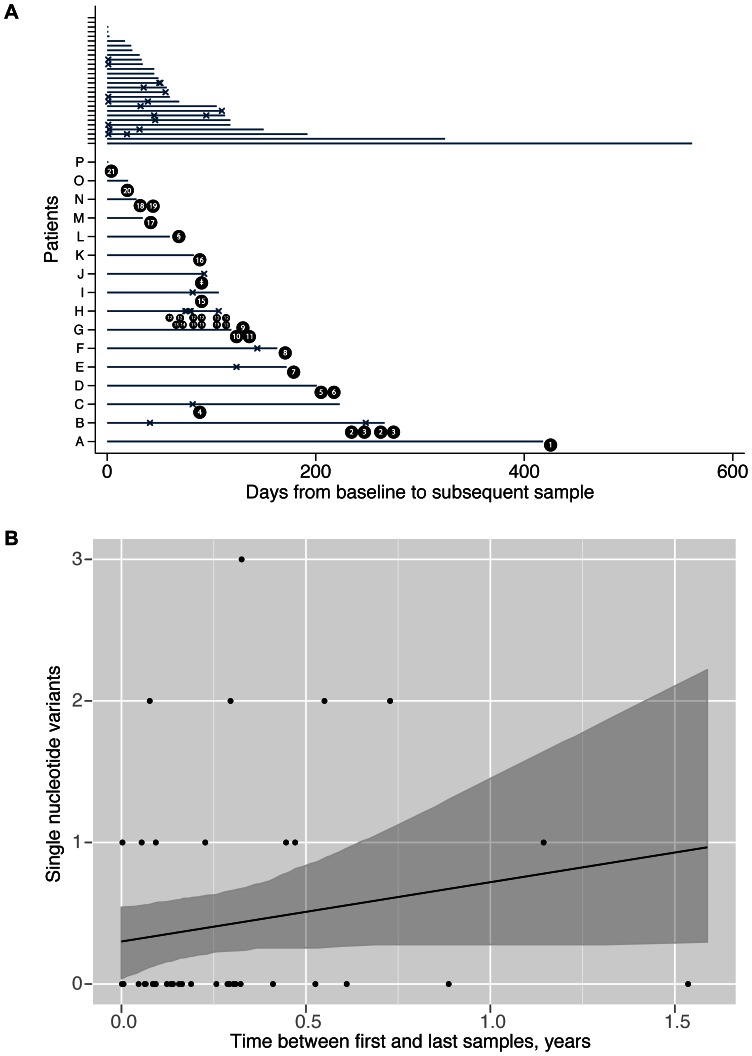
Observed single nucleotide variants between serially sampled patients with ST1/ribotype 027 CDI. Panel A shows each patient as a separate horizontal line. Samples were obtained from each patient at the start and end of each line, any additional intermediate samples obtained are shown with a ‘x’. Twenty-eight patients with no SNVs at any timepoint are shown at the top of the plot. The 16 patients with at least 1 SNV observed at ≥1 timepoint are lettered A to P. SNVs are shown as black circles, the number corresponding to table 1. ‡ indicates a patient with 13 transient SNVs, observed after 93 days, and with a sample indistinguishable from baseline at 94 days. § indicates a patient with 20 SNVs presumed to be a re-infection, and excluded from panel B. Panel B shows the time between first and last samples from 43 serially sampled patients. The line indicates a coalescent-based model for the rate of *C. difficile* evolution fitted by maximum likelihood assuming a Poisson distribution for the accumulation of mutations. 95% confidence intervals obtained by parametric bootstrap are shown shaded.

A total of 122 isolates were obtained from 122 faecal samples from 44 patients with mostly recurrent, or on-going, CDI due to *C. difficile* ribotype 027/ST1 (23 patients with 2 samples, 12 patients with 3 samples, 8 patients with 4 samples and 1 patient with 8 samples). The median (IQR, range) time between first and last samples was 60 (29.5–118.5, 0–561) days. A single patient with 20 SNVs between first and last samples taken 60 days apart was excluded because the subsequent sample was considered more likely to represent a re-infection, as there was no evidence of a single recombination, no SNVs in DNA mismatch repair genes, and a sequence matching the later sample had previously been found in several other patients admitted to an adjacent ward at the same time. In the remaining 43 patients, successive *C. difficile* isolates from 28 patients had no SNVs. A further 3 patients had isolates with transient SNVs that were not detected at the final timepoint: 2 had a single SNV, and the other had 13 SNVs distributed throughout the genome after 93 days; in the latter case, however, the isolate recovered from a sample collected on the following day was identical to the initial isolate, suggesting a mixed infection in the transient 13 SNV isolate. The remaining patients had isolates with 1 SNV (n = 7), 2 SNVs (n = 4) or 3 SNVs (n = 1) (Figure 5a) at the final timepoint. All SNVs were distinct from those observed in the gut model. The overall rate of evolution estimated from first and last samples from these 43 serially sampled patients was 0.42 SNVs/year (95% CI 0.00–1.30) in the sites routinely called by sequencing (mean 90.0% of the CD196 reference genome) (Figure 5b). The within-patient diversity was 0.28 SNVs/called genome (95% CI 0.04–0.54). Excluding a single out-lying point at 561 days with 0 SNVs in a sensitivity analysis, the rate of evolution was 0.75 (95%CI 0.00–1.87)/called genome/year and the within-patient diversity 0.16 SNVs/called genome (95%CI 0.00–0.46).

### Functional Impact of Variants

The functional impact of SNVs arising in the gut model isolates and serial sample isolates from infected patients is shown in table 1. Nine of the 10 SNVs across the three gut models occurred within an annotated coding sequence, and all resulted in a non-synonymous change, including two causing a premature stop codon. Similarly 19 of the 21 SNVs from patient samples occurred in coding sequence, with only 2 resulting in synonymous changes, and the remainder non-synonymous, including 4 premature stop codons. No patients developed the same SNV independently. There was only one occurrence of the same gene being affected by two mutations, both occurring in a RNA polymerase sigma-B factor gene in the co-amoxiclav induction gut model. Transcription and transport/metabolism pathways were affected by non-synonymous SNVs in both clinical and gut model isolates.

**Table 1:** Identified SNVs in gut model and patient *C. difficile* isolates.

Gut Model	COG Function		SNV site		Locus	Wildtype	SNV	Impact	Annotation	Fig
	Carbohydrate transport and metabolism		2914411		CD196_2507	C	T	Truncated	PTS system, glucose-specific IIa component	4
	Carbohydrate transport and metabolism		3391317		CD196_2881	G	T	Non-Synonymous	PTS system, IIabc component	2,3
	Transcription		9921		CD196_0011	A	G	Non-Synonymous	RNA polymerase sigma-B factor	2
	Transcription		10179		CD196_0011	C	T	Non-Synonymous	RNA polymerase sigma-B factor	2
	Poorly characterized		977041		CD196_0822	C	T	Non-Synonymous	ABC transporter, substrate-binding lipoprotein	3
	Poorly characterized		2380323		CD196_2054	C	T	Non-Synonymous	putative phosphoesterase	3
	Poorly characterized		3493190		CD196_2947	A	G	Non-Synonymous	crispr-associated helicase cas3	3
	Poorly characterized		1592378		CD196_1367	G	T	Truncated	putative transcriptional regulator	2,3
	**–**		2206108		CD196_1916	T	C	Non-Synonymous	putative lipoprotein	3
	There was 1 SNV, not within an annotated coding region: 2916358									2,3
**Patients**	**COG function**	**SNV site**		**Locus**		**Wildtype**	**SNV**	**Impact**	**Annotation**	**Legend**
	Amino acid transport and metabolism	444759		CD196_0382		C	A	Non-Synonymous	subunit of oxygen-sensitive 2-hydroxyisocaproyl-CoA dehydratase	15
	Amino acid transport and metabolism	584702		CD196_0499		G	T	Non-Synonymous	glutaminase	18
	Amino acid transport and metabolism	2546482		CD196_2190		G	T	Non-Synonymous	putative Xaa-Pro dipeptidase	3
	Cell envelope biogenesis, outer membrane	3348892		CD196_2850		C	T	Non-Synonymous	hypothetical protein	4
	Inorganic ion transport and metabolism	2268756		CD196_1966		C	T	Non-Synonymous	putative Na(+)/H(+) antiporter	8
	Inorganic ion transport and metabolism	3645413		CD196_3074		G	T	Truncated	putative phosphateABC transporter, permease protein	10
	Posttranslational modification, protein turnover, chaperones	1878408		CD196_1614		C	T	Non-Synonymous	thioredoxin reductase	21
	Posttranslational modification, protein turnover, chaperones	3073073		CD196_2629		C	A	Non-Synonymous	cell surface protein (putative cell surface-associated cysteine protease)	17
	Signal transduction mechanisms	1989705		CD196_1719		G	T	Truncated	tellurium resistance protein	9
	Signal transduction mechanisms	2023590		CD196_1748		C	A	Non-Synonymous	two-component sensor histidine kinase	2
	Signal transduction mechanisms	3037826		CD196_2601		C	A	Truncated	putative signaling protein	14
	Signal transduction mechanisms	4091272		CD196_3472		G	T	Non-Synonymous	putative RNA/single-stranded DNA exonuclease	11
	Transcription	1378004		CD196_1170		A	G	Synonymous	transcription elongation protein	16
	Transcription	2886178		CD196_2484		G	A	Non-Synonymous	sporulation sigma factor SigE	1
	Transcription	2970564		CD196_2552		C	T	Truncated	LysR-family transcriptional regulator	6
	Poorly characterized	3269840		CD196_2784		G	T	Non-Synonymous	hypothetical protein	13
	Poorly characterized	3244418		CD196_2762		C	T	Synonymous	putative acetyltransferase	12
	–	2263760		CD196_1962		A	C	Non-Synonymous	hypothetical protein	19
	–	3561141		CD196_3004		G	A	Non-Synonymous	abc transporter, permease associated with salivaricin lantibiotic	7
	There were 2 SNVs, not within an annotated coding region: 783919, 797285									20,5

COG, Clusters of Orthologous Groups of proteins. Fig refers to the figure displaying the SNV. Legend refers to the SNV identifiers used in figure 5.

## Discussion

We have demonstrated using isolates obtained from two complimentary sources, recurrent CDI in patients and a predictive experimental gut model of CDI, that there is a high degree of *C. difficile* genome stability as measured by WGS. The two investigated scenarios represented opportunities for *C. difficile* genome mutation occurring over periods of one to several months. The studied settings represent relatively controlled and uncontrolled stresses that could be expected to increase the chance of SNV occurrence. Both yielded isolates from CDI symptomatic/toxin production periods and after treatment.

Both the gut model and clinical patient isolates showed evidence of within host variation at the initial sample, either directly in the case of the gut model, or from the evolutionary model in the clinical samples. This contributed significantly to SNV differences observed between baseline and subsequent samples. The 2 new SNVs observed over 137 days of gut model follow up are consistent with the estimates for *C. difficile* evolution of 0.42 (95% CI 0.00–1.30) SNVs/sampled genome/year obtained from serially sampled patients. The relatively broad confidence interval, encompassing zero, reflects the limited number of recurrent ribotype 027 infections in our dataset, and is a limitation of our study. When a single outlier with zero SNVs after 561 days was removed, the estimated rate of evolution increased modestly to 0.75 (95%CI 0.00–1.87) SNVs/called genome/year. This highlights the potential difficulties in applying a single clock rate to *C. difficile*, as the prolonged time with zero SNVs could represent time spent dormant in a spore state with a slower rate of evolution, which would lower the overall estimated rate of evolution. The estimates obtained are similar to previous estimates where data from ribotype 027 and non-ribotype 027 samples were combined. [16].

The presence of minority variants detected by sequencing sub-cultures of multiple colonies, whilst not unexpected, is a reminder that diversity may be detected between transmitted cases as a result of sampling a minority variant in either the donor or the recipient. Similarly, if only a single colony is sub-cultured, potential mixed infection may also be missed. However sequencing sweeps of primary culture plates using existing data pipelines may also miss minority variants if these are attributed to sequencing error, or result in a heterozygous base calls, which may be converted to a null call by sequencing quality filtering algorithms. Therefore, sequencing the full diversity present on primary culture plates together with bioinformatic approaches specifically designed to detect mixed infection may provide an optimal approach [24].

While relatively few mutations were observed, a striking proportion were non-synonymous. Twenty-six of 28 SNVs in coding regions were non-synonymous, with 6 resulting in premature truncation of a gene. This corresponds to a dN/dS ratio (ratio of non-synonymous vs. synonymous substitution rate, adjusted for the redundancy in the codons affected) of 5.3 (95% CI 1.6 to ∞), which contrasts with the dN/dS ratio reported in another whole genome study of recently diverged *C. difficile* lineages of very close to 1. [25] However the genomes in this previous study were separated by up to several years, rather than several weeks as was typical in our study. Although dN/dS ratios above 1 are suggestive of SNVs arising in response to selective pressure, such as might be experienced during antibiotic exposure, short-term ratios are typically higher owing to a lag in the removal of slightly deleterious mutations. [26] A number of transient SNVs were also observed, consistent with genetic drift. Whilst the classification of genes with SNVs is intriguing, with SNVs arising after antibiotic exposure in genes involved in transcription and transport/metabolism pathways, much larger formal association studies are required to understand their functional significance.

Another limitation of our study is that we do not have details of the specific interventions, particularly treatments, for individual CDI cases. However, during the study hospital policy was to treat initial CDI and first recurrence with oral vancomycin for 14 days; treatment was initiated when samples were sent for *C. difficile* testing and compliance was high (data not shown). Where patient’s first and subsequent samples were more than 14 days apart (63 of the 78 subsequent samples obtained), we have assumed that these represent recurrences of CDI (in these cases, relapses with the same strains), or possibly failure to respond to initial treatment in some patients. Given the prolonged observation periods, it is highly likely that some of the strains we have examined were subjected to multiple exposures to antibiotics used to treat CDI and possibly other infections, and this could be explored in more detail in future studies. In common with the patients studied, CDI treatment with vancomycin was also simulated in one of the gut models. Whilst it would also be of interest to repeat the study in a population receiving metronidazole treatment (not used in Oxfordshire hospitals), and to simulate metronidazole treatment, the consistency of the results obtained to date suggest that this would be unlikely to materially alter our findings. The antibiotic used to treat simulated CDI in the final gut model is a hybrid molecule, which is currently in phase II clinical trials for the treatment of CDI. [14], [15].

Recombination and hypermutation are two ways in which the modest rate of genome mutation we observed here could in theory accelerate. Hypermutation is not well studied in *C. difficile*. In this study, we found no variant sites arising within annotated DNA mismatch repair enzymes, responsible for hypermutation described in *Staphylococcus aureus* [27] and other pathogens. [28] There is clear evidence for recombination in the *C. difficile* genome over longer timescales, [3], [16], [25] although interestingly it is not seen as frequently in ribotype 027 isolates compared to some other lineages. [16] With relatively few variants and short time scales we did not find evidence of clustering of nearby variable sites suggestive of recombination in our study. However, the absence of *C. difficile* mixed infection in the gut model precludes within-species recombination.

Our data showing relatively stable genomes, and low but non-zero microevolution, in the face of interventions and indeed repeated episodes of infection and treatment provide reassurance that WGS can be used to (re-) examine the epidemiology of *C. difficile* and aetiology of CDI. For example, using genotyping (MLST) we recently unexpectedly found that about three-quarters of CDI cases could not be linked to other cases by ward-based contact, [5] raising the issue of other sources of *C. difficile* transmission not well addressed by current infection control policy and guidance. Whole genome sequence data offer the potential to study such *C. difficile* transmission in more detail. [16] To interpret WGS data, however, it is necessary to first establish the ‘natural’ rate of genome mutation. Without such microevolutionary information, use of such a highly discriminatory fingerprinting method risks dismissing CDI cases and sources that could in fact be related. [9] Speculation about the aetiology of community CDI cases and indeed the possible links between human and animal *C. difficile* strains emphasise the need for optimised fingerprinting studies. [29], [30] A better understanding of other possible routes of transmission and reservoirs is needed to optimise control efforts.

In summary, the consistency of sequences from our gut model isolates demonstrates the reliability of our sequencing and analysis techniques. Crucially, the high stability of mapped genomic sequences in *C. difficile* isolates from patients supports their use in detailed transmission studies. Our findings also highlight the importance of detailed microevolutionary studies specific to key pathogens to maximise the future value of pathogen whole genome sequencing.

### Data Sharing

The sequences reported in this paper have been deposited in the European Nucleotide Archive Sequence Read Archive under study accession number ERP002404: Short-term stability of C. difficile 027 isolates in a gut model and recurrent disease.

## Supporting Information

Text S1 **Description of coalescent-based model used to estimate within-host diversity and rate of** ***C. difficile*** **evolution.**
(DOCX)

## References

[1] Killgore G, Thompson A, Johnson S, Brazier J, Kuijper E, et al (2008) Comparison of seven techniques for typing international epidemic strains of Clostridium difficile: restriction endonuclease analysis, pulsed-field gel electrophoresis, PCR-ribotyping, multilocus sequence typing, multilocus variable-number tandem-repeat analysis, amplified fragment length polymorphism, and surface layer protein A gene sequence typing. J Clin Microbiol 46: 431–437 doi:10.1128/JCM.01484-07. 18039796 10.1128/JCM.01484-07 PMC2238077

[2] Griffiths D, Fawley W, Kachrimanidou M, Bowden R, Crook DW, et al (2010) Multilocus sequence typing of Clostridium difficile. J Clin Microbiol 48: 770–778 doi:10.1128/JCM.01796-09. 20042623 10.1128/JCM.01796-09 PMC2832416

[3] Dingle KE, Griffiths D, Didelot X, Evans J, Vaughan A, et al (2011) Clinical Clostridium difficile: clonality and pathogenicity locus diversity. PLoS ONE 6: e19993 doi:10.1371/journal.pone.0019993. 21625511 10.1371/journal.pone.0019993 PMC3098275

[4] Lemée L, Dhalluin A, Pestel-Caron M, Lemeland J-F, Pons J-L (2004) Multilocus sequence typing analysis of human and animal Clostridium difficile isolates of various toxigenic types. J Clin Microbiol 42: 2609–2617 doi:10.1128/JCM.42.6.2609-2617.2004. 15184441 10.1128/JCM.42.6.2609-2617.2004 PMC427854

[5] Walker AS, Eyre DW, Wyllie DH, Dingle KE, Harding RM, et al (2012) Characterisation of Clostridium difficile hospital ward-based transmission using extensive epidemiological data and molecular typing. PLoS Med 9: e1001172 1–e1001172: 12 doi:10.1371/journal.pmed.1001172. 10.1371/journal.pmed.1001172 PMC3274560 22346738

[6] Freeman J, Bauer MP, Baines SD, Corver J, Fawley WN, et al (2010) The Changing Epidemiology of Clostridium difficile Infections. Clin Microbiol Rev 23: 529–549 doi:10.1128/CMR.00082-09. 20610822 10.1128/CMR.00082-09 PMC2901659

[7] Wilcox MH, Shetty N, Fawley WN, Shemko M, Coen P, et al (2012) Changing Epidemiology of Clostridium difficile Infection Following the Introduction of a National Ribotyping-Based Surveillance Scheme in England. Clin Infect Dis 55: 1056–1063 doi:10.1093/cid/cis614. 22784871 10.1093/cid/cis614

[8] Didelot X, Bowden R, Wilson DJ, Peto TEA, Crook DW (2012) Transforming clinical microbiology with bacterial genome sequencing. Nat Rev Genet 13: 601–612 doi:10.1038/nrg3226. 22868263 10.1038/nrg3226 PMC5049685

[9] Eyre DW, Golubchik T, Gordon NC, Bowden R, Piazza P, et al (2012) A pilot study of rapid benchtop sequencing of Staphylococcus aureus and Clostridium difficile for outbreak detection and surveillance. BMJ Open 2: e001124 doi:10.1136/bmjopen-2012–001124. 10.1136/bmjopen-2012-001124 PMC3378946 22674929

[10] Köser CU, Holden MTG, Ellington MJ, Cartwright EJP, Brown NM, et al (2012) Rapid whole-genome sequencing for investigation of a neonatal MRSA outbreak. N Engl J Med 366: 2267–2275 doi:10.1056/NEJMoa1109910. 22693998 10.1056/NEJMoa1109910 PMC3715836

[11] Baines SD, O’Connor R, Saxton K, Freeman J, Wilcox MH (2009) Activity of vancomycin against epidemic Clostridium difficile strains in a human gut model. J Antimicrob Chemother 63: 520–525 doi:10.1093/jac/dkn502. 19112083 10.1093/jac/dkn502

[12] Best EL, Freeman J, Wilcox MH (2012) Models for the study of Clostridium difficile infection. Gut Microbes 3: 145–167 doi:10.4161/gmic.19526. 22555466 10.4161/gmic.19526 PMC3370947

[13] Chilton CH, Freeman J, Crowther GS, Todhunter SL, Nicholson S, et al (2012) Co-amoxiclav induces proliferation and cytotoxin production of Clostridium difficile ribotype 027 in a human gut model. J Antimicrob Chemother 67: 951–954 doi:10.1093/jac/dkr584. 22279183 10.1093/jac/dkr584

[14] Baines SD, Crowther GS, Todhunter SL, Freeman J, Wilcox MH (2012) *In vitro* activity of cadazolid (act-179811) against *Clostridium difficile* and in an *in vitro* gut model of *C. difficile* infection [Abstract B-662]. In: Program and abstracts of 52nd Interscience Conference on Antimicrobial Agents and Chemotherapy (San Francisco).

[15] Baldoni D, Gutierrez M, Dingemanse J, Timmer W (2012) Cadazolid, a novel antibiotic with potent activity against *Clostridium difficile*: safety, tolerability, and pharmacokinetics in healthy subjects following single and multiple oral doses [Abstract A-1273]. In: Program and abstracts of 52nd Interscience Conference on Antimicrobial Agents and Chemotherapy (San Francisco). 10.1093/jac/dkt401 24106141

[16] Didelot X, Eyre DW, Cule ML, Ip CL, Ansari MA, et al (2012) Microevolutionary analysis of Clostridium difficile genomes to investigate transmission. Genome Biol 13: R118 doi:10.1186/gb-2012-13-12-r118. 23259504 10.1186/gb-2012-13-12-r118 PMC4056369

[17] Loman NJ, Constantinidou C, Chan JZM, Halachev M, Sergeant M, et al (2012) High-throughput bacterial genome sequencing: an embarrassment of choice, a world of opportunity. Nat Rev Microbiol 10: 599–606 doi:10.1038/nrmicro2850. 22864262 10.1038/nrmicro2850

[18] Lunter G, Goodson M (2011) Stampy: A statistical algorithm for sensitive and fast mapping of Illumina sequence reads. Genome Res 21: 936–939 doi:10.1101/gr.111120.110. 20980556 10.1101/gr.111120.110 PMC3106326

[19] Stabler RA, He M, Dawson L, Martin M, Valiente E, et al (2009) Comparative genome and phenotypic analysis of Clostridium difficile 027 strains provides insight into the evolution of a hypervirulent bacterium. Genome Biol 10: R102 doi:10.1186/gb-2009-10-9-r102. 19781061 10.1186/gb-2009-10-9-r102 PMC2768977

[20] Li H, Handsaker B, Wysoker A, Fennell T, Ruan J, et al (2009) The Sequence Alignment/Map format and SAMtools. Bioinformatics 25: 2078–2079 doi:10.1093/bioinformatics/btp352. 19505943 10.1093/bioinformatics/btp352 PMC2723002

[21] Altschul SF, Madden TL, Schäffer AA, Zhang J, Zhang Z, et al (1997) Gapped BLAST and PSI-BLAST: a new generation of protein database search programs. Nucleic Acids Res 25: 3389–3402. 9254694 10.1093/nar/25.17.3389 PMC146917

[22] Rodrigo AG, Felsenstein J (1999) Coalescent approaches to HIV population genetics. In: Crandall KA, editor. Evolution of HIV. Baltimore, MD: Johns Hopkins University Press. pp. 233–272.

[23] Nei M, Gojobori T (1986) Simple methods for estimating the numbers of synonymous and nonsynonymous nucleotide substitutions. Mol Biol Evol 3: 418–426. 3444411 10.1093/oxfordjournals.molbev.a040410

[24] Eyre DW, Cule ML, Griffiths D, Crook DW, Peto TEA, et al. (2013). Detection of mixed infection from bacterial whole genome sequence data allows assessment of its role in *Clostridium difficile* transmission. PLOS Comput Biol [In Press]. 10.1371/journal.pcbi.1003059 PMC3642043 23658511

[25] He M, Sebaihia M, Lawley TD, Stabler RA, Dawson LF, et al (2010) Evolutionary dynamics of Clostridium difficile over short and long time scales. Proc Natl Acad Sci USA 107: 7527–7532 doi:10.1073/pnas.0914322107. 20368420 10.1073/pnas.0914322107 PMC2867753

[26] Rocha EPC, Smith JM, Hurst LD, Holden MTG, Cooper JE, et al (2006) Comparisons of dN/dS are time dependent for closely related bacterial genomes. J Theor Biol 239: 226–235 doi:10.1016/j.jtbi.2005.08.037. 16239014 10.1016/j.jtbi.2005.08.037

[27] Prunier AL, Leclercq R (2005) Role of mutS and mutL Genes in Hypermutability and Recombination in Staphylococcus aureus. J Bacteriol 187: 3455–3464 doi:10.1128/JB.187.10.3455-3464.2005. 15866932 10.1128/JB.187.10.3455-3464.2005 PMC1112015

[28] Oliver A, Mena A (2010) Bacterial hypermutation in cystic fibrosis, not only for antibiotic resistance. Clin Microbiol Infect 16: 798–808 doi:10.1111/j.1469-0691.2010.03250.x. 20880409 10.1111/j.1469-0691.2010.03250.x

[29] Hensgens MPM, Keessen EC, Squire MM, Riley TV, Koene MGJ, et al (2012) Clostridium difficile infection in the community: a zoonotic disease? Clin Microbiol Infect 18: 635–645 doi:10.1111/j.1469-0691.2012.03853.x. 22536816 10.1111/j.1469-0691.2012.03853.x

[30] Bakker D, Corver J, Harmanus C, Goorhuis A, Keessen EC, et al (2010) Relatedness of human and animal Clostridium difficile PCR ribotype 078 isolates determined on the basis of multilocus variable-number tandem-repeat analysis and tetracycline resistance. J Clin Microbiol 48: 3744–3749 doi:10.1128/JCM.01171-10. 20686080 10.1128/JCM.01171-10 PMC2953124

