# Supplementary material for: Short-Term Genome Stability of Serial Clostridium difficile Ribotype 027 Isolates in an Experimental Gut Model and Recurrent Human Disease
Source: PLoS One. 2013 May 15;8(5):e63540. doi: 10.1371/journal.pone.0063540 (PMC3655144; doi:10.1371/journal.pone.0063540)
Supplement: Text S1 — Description of coalescent-based model used to estimate within-host diversity and rate of C. difficile evolution. (DOCX) [file pone.0063540.s001.docx]

**Short-term genome stability of serial *Clostridium difficile* ribotype 027 isolates in an experimental gut model and recurrent human disease**

Eyre DW, Walker AS, Freeman J *et al*

**Text S1**

The within-host diversity and rate of *C. difficile* evolution was estimated by maximum likelihood from first and last isolates from serially sampled patients under a coalescent model assuming a Poisson distribution for the accumulation of mutations (1).

The amount of diversity observed between a pair of samples obtained at times t_0_ and t_1_ can be thought of as the sum of 2 Poisson processes, firstly the variation that arose in the time, *t*, between the samples being taken, and secondly the variation that has arose in the time, *u*, between the most recent common ancestor of the two samples and time t_0_ (Figure).

**Figure. Coalescent-based model.** t_0_ denotes the time of the first sample, t_1_ the time of the second sample. *t* is the time between the samples, and *u* the time between the first sample and the common ancestor of the samples.

The sum of the two processes is itself a Poisson process, however the value of *u* is unknown. Given a constant rate of evolution, *µ,* and for a given value of *u,* the number of single nucleotide variants between the samples, *s*, is given by:

$$s|u \sim Pois(\mu t)+Pois(\mu2u)$$

Which can be written:

$$s|u \sim Pois(\mu t+\mu2u)$$

Under coalescent theory, with neutral selection and a fixed population size, *u* is exponentially distributed with mean *N_e_*, the effective population size:

$$u \sim Exp(1/N_{e})$$

The value of *N_e_* and *µ* can be jointly estimated by maximum likelihood. To do this the likelihood of *N_e_* and *µ* given the data is defined as follows. Firstly the probability mass function for $s|u$ can be written:

$$\Pr\left( s | u \right)= \frac{e^{-\mu(t+2u)}{(\mu(t+2u))}^{s}}{s!}$$

The probability of *s*, unconditional on *u*, i.e. the likelihood of *µ* and *N_e_* for a single observation, can be expressed by integrating over all possible values of *u*:

$$\Pr\left( s \right)= \int_{0}^{\infty} \Pr\left( s | u \right)\frac{1}{N_{e}}e^{-\frac{1}{N_{e}}u}\mathrm{du}$$

$$\Pr\left( s \right)= \frac{e^{-\mu t}}{N_{e}s!}\int_{0}^{\infty} {(\mu(t+2u))}^{s}e^{-u(2\mu+\frac{1}{N_{e}})}\mathrm{du}$$

Maple (<http://www.maplesoft.com/products/maple/>) was used to solve the integral. The data can be thought of as *n* observed pairs of samples separated by time *t_i_* and with single nucleotide variants *s_i_* between them. Therefore to calculate the likelihood of *µ* and *N_e_* given the data, the product of the Pr(*s_i_*) across all the *n* pairs of data was calculated:

$$L(\mu,N_{e} | data)= \prod_{i=1}^{i=n} \Pr(s_{i})$$

Maximum likelihood values of *µ* and *N_e_* were found by numerical optimisation using R (<http://www.r-project.org>). 95% confidence intervals for parameter estimates were calculated by parametric bootstrap, using 1000 iterations.

**References**

1. **Rodrigo AG**, **Felsenstein J**. 1999. Coalescent approaches to HIV population genetics, pp. 233–272. *In* Crandall, KA (ed.), Evolution of HIV. Johns Hopkins University Press, Baltimore, MD.
